# Supplementary material for: Real-time high-resolution mid-infrared optical coherence tomography
Source: Light Sci Appl. 2019 Jan 23;8:11. doi: 10.1038/s41377-019-0122-5 (PMC6342823; doi:10.1038/s41377-019-0122-5)
Supplement: Supplementary file 1 — Supplementary Material [file 41377_2019_122_MOESM1_ESM.docx]

1. **Samples**

Real-time High-resolution Mid-infrared Optical Coherence Tomography

Niels M. Israelsen^1,7^, Christian R. Petersen^1,7,*^, Ajanta Barh^2^, Deepak Jain^1^, Mikkel Jensen^1^, Günther Hannesschläger^3^, Peter Tidemand-Lichtenberg^2,5^, Christian Pedersen^2,5^, Adrian Podoleanu^4^ and Ole Bang^1,6,7^

^1^ DTU Fotonik, Technical University of Denmark, DK-2800 Kgs. Lyngby, Denmark
^2^ DTU Fotonik, Technical University of Denmark, DK-4000 Roskilde, Denmark
^3^ Research Center for Non-Destructive Testing (RECENDT), Altenberger Straße 69, 4040 Linz, Austria
^4^ Applied Optics Group, School of Physical Sciences, University of Kent, CT2 7NH Canterbury, United Kingdoms
^5^ NLIR ApS, Hirsemarken 1, 3520 Farum, Denmark
^6^ NKT Photonics A/S, Blokken 84, DK-3460 Birkerød, Denmark
^7^ NORBLIS IVS, Virumgade 35 D, 2830 Virum, Denmark

**Supplementary MATERIAL**

**Ceramics**

Figure S1 shows a photograph of the ceramic plates together with *en face* microscopy and OCT images of the largest and smallest features in the microstructured alumina sample.


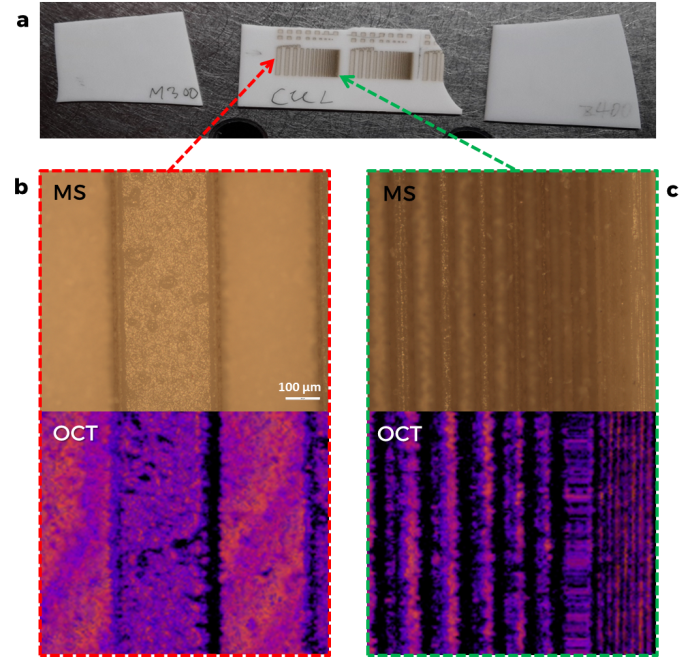


Fig. S1 Images of the ceramic stack. a Photograph of the three ceramic plates (Left: alumina plate, middle: laser-milled alumina plate, right: zirconia plate) . b,c Microscopy (MS) and OCT images of the largest and smallest features of the laser-milled ceramic sample. OCT images were obtained through 775 μm of alumina.

**Credit card**

The credit card used for imaging is a standard VISA card issued by a Danish bank. Fig. S2a shows a photograph of the credit card with a dashed line indicating the position of a single B-scan cut performed for both OCT systems. The ink from the letter “E” is seen in the 1.3 μm OCT image as three shadows, and in the 4 μm OCT image as three bright lines.

Fig. S2b shows a cross-sectional view of the credit card (top) together with four *en face* images (P1-P4) corresponding to different layers in the volume.

**^
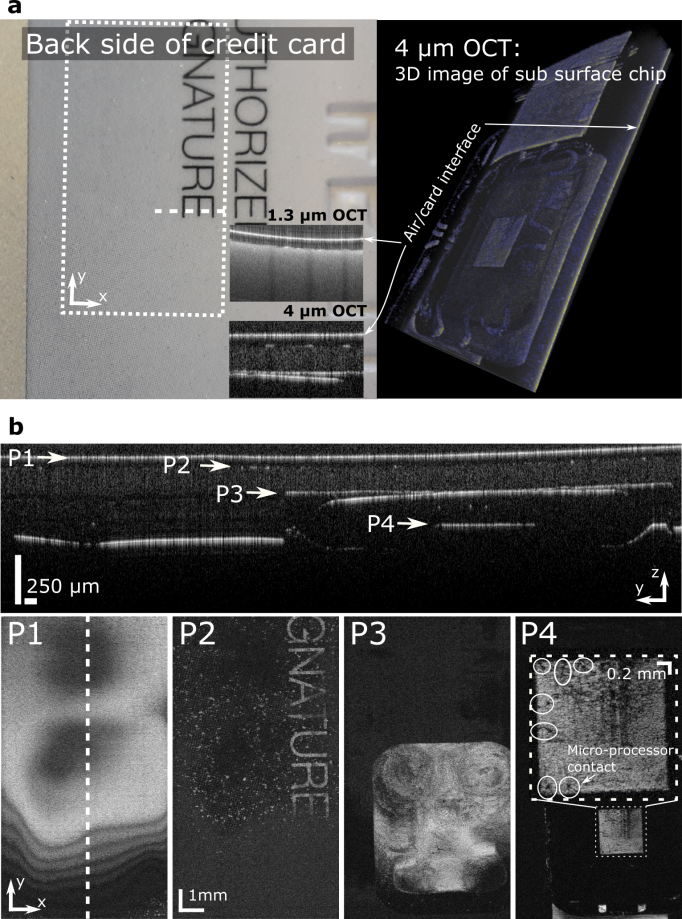
^**

Fig. S2 4 µm OCT images in relation to real color photo of a standard credit card. a Photograph of the backside scanned credit card (left). Insets display B-scan cuts (along the white dashed line) obtained using the two OCT systems. The dotted rectangle embraces the volume presented in the 3D visualization (right), showing sub-surface elements of the credit card , and in b. b B-scan map (top) with marked *en face* cuts (P1-P4). Dashed line in P1 marks the B-scan cut in b.

1. **Supplementary videos**

Four supplementary videos are available:

1. Video of Monte-Carlo simulations for 1.3 μm vs. 4 μm (**MonteCarlo.avi**)


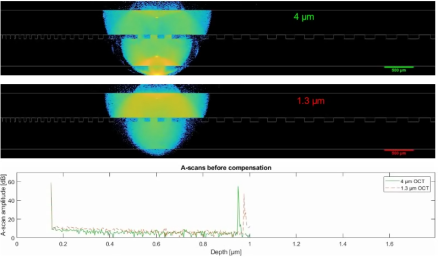


1. Video of ceramic stack imaged from the top (**CeramicStackTop.avi**).


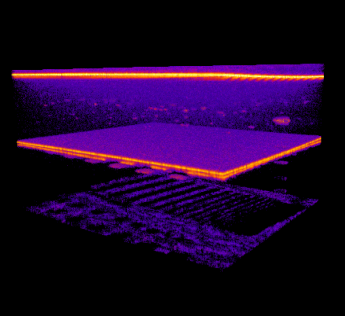


1. Video of ceramic stack imaged from the bottom (**CeramicStackBottom.avi**).


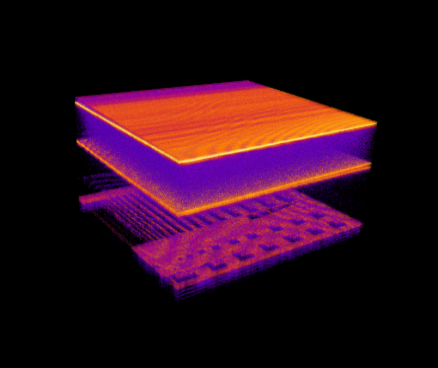


1. Video of credit card (**CreditCard.avi**).


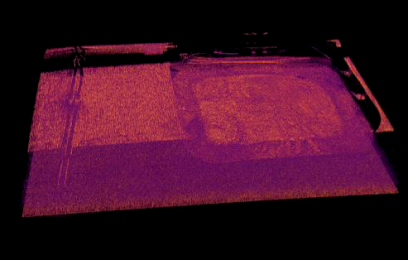


1. **Upconversion Details**

In the parametric frequency upconversion process, all three waves, pump, input mid-infrared (mid-IR) and upconverted, are considered as extraordinarily polarized and they satisfy momentum conservation through non-collinear interaction inside the periodically poled lithium niobate (PPLN) crystal. The pump beam (1064 nm) passes straight through the PPLN, whereas the input mid-IR signal is focused inside it (Fig. S3a). For a certain poling period (Λ) of the PPLN, a photon of given mid-IR wavelength travelling at certain angle with the pump can generate a corresponding upconverted photon efficiently. The dependence of phase-matched mid-IR wavelengths vs. angles is plotted in Fig. S3b. The upconverted photons are generated at corresponding angles symmetrically around the pump axis, producing a donut shaped beam at the output (see Fig. S4). By choosing different Λ (meaning different grating vector, k_Λ_), the phase-matching bandwidth can be tailored (Fig. S3b). For the 4 µm OCT system, Λ = 23 µm was chosen. The quantum efficiency (QE) is calculated by multiplying power conversion efficiency with the ratio of wavelengths (upconverted/mid-IR), see Fig. S3c. The gradual decrease in QE with increasing wavelength is mainly due to the increasing linear absorption loss of PPLN at longer mid-IR wavelengths.

The schematic diagram of the intra-cavity laser based upconversion module is shown in Fig.S4.

**(a)**

**(b)**


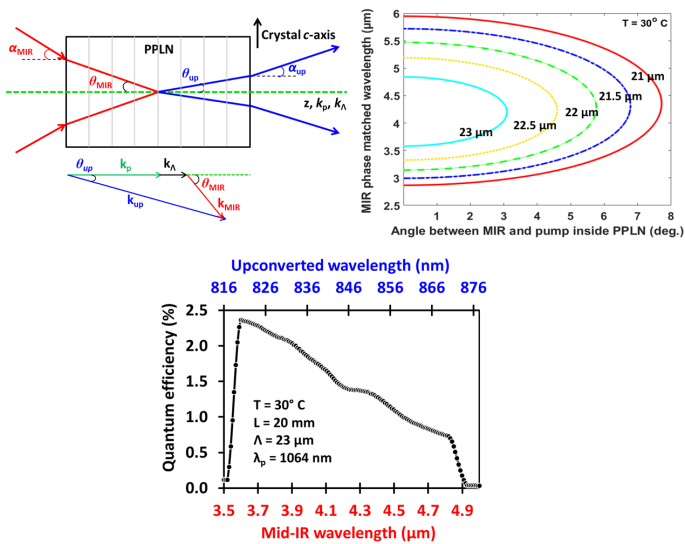


**(c)**

Fig. S3 Characteristics of frequency upconversion. a Schematic representation of propagation directions of the pump, mid-IR and upconversion wavevectors (k_p_, k_MIR_, k_up_) through the PPLN. The z-axis and pump bam are along the crystal length. b Phase-matched wavelength versus angle for different values of the PPLN poling period (21, 21.5, 22, 22.5, 23 µm). c Variation of the quantum efficiency of the upconversion process versus mid-IR wavelength; the upconverted wavelength is shown along the horizontal top axis.


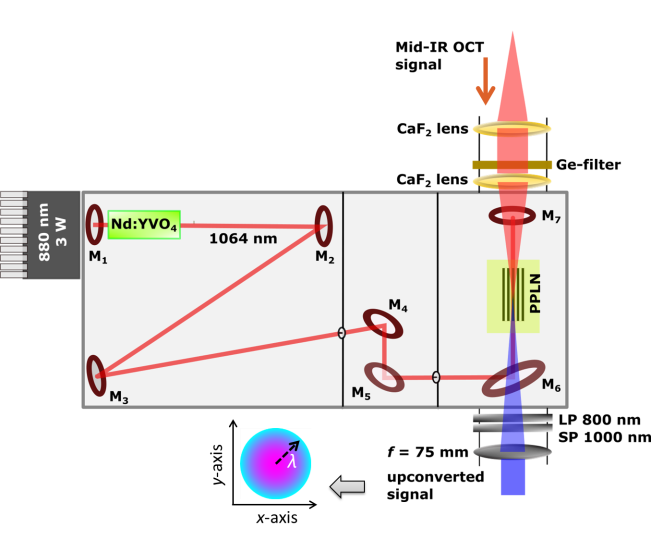


Fig. S4 Schematic of the frequency upconversion module. The PPLN is placed inside the 1064 nm pump laser cavity to gain access to the highest pump power. Ge-filter is used to block any incoming light of wavelength below 2 µm. M1-M7: HR-coated mirrors at 1064 nm; M6: AR coated at upconverted wavelength; M7: HT-coated for 2 – 5 µm range. LP: low pass filter; SP: short pass filter. The radial wavelength distribution across the transverse area of the upconverted beam is shown.

1. **Description of the 1.3 µm system**

The 1.3 µm OCT system used as a benchmark for comparison of its images with those produced by the 4 µm OCT system is sketched in Fig. S5. A 320 MHz supercontinuum source (SuperK Extreme EXR-9) from NKT Photonics, Denmark, providing broadband light, is bandpass filtered to the wavelength range of 1-1.5 µm. The light is coupled to a four-arm fiber coupler from Gooch and Housego, UK, acting as a Michelson Interferometer with a free-space reference and sample arm built from simple interface optics. The mixed signal of the reference and sample arm is guided to and detected with a 2048 pixel spectrometer covering the range of 1074-1478 nm. Calibration and dispersion compensation are performed digitally. For further details we refer to ref. 24 in the manuscript.


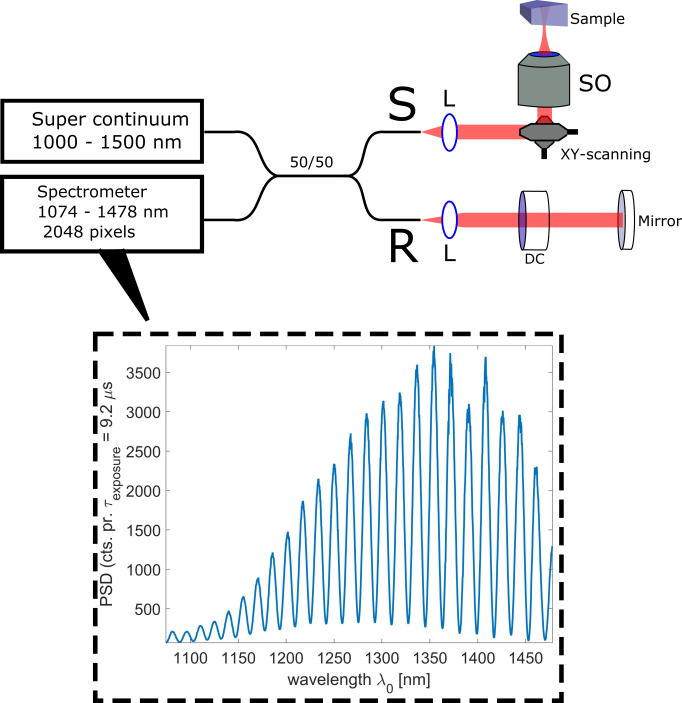


Fig. S5 Schematic of the 1.3 µm OCT system setup. Main components are a supercontinuum source, fiber coupler (50/50), spectrometer, sample (S), and reference (R). S and R encompass collimating lenses (L), galvonometric scanners (XY), scanning objective (SO), dispersion compensating element (DC). The graph shows an example of an interferogram of a channeled spectrum.

1. **Comparing 1.3 µm and 4 µm OCT images**

The sensitivity roll-off curves versus one-way optical path difference (OPD) are presented in Fig. S6. The signal attenuation with OPD is seen to be similar for the two systems, with a maximum deviation of 3 dB between their roll-offs over a 2 mm OPD axial range. The logarithmic slopes are also similar.

The moderate attenuation slope with OPD allows tolerance in setting the position of focus in the sample, as well as sample positioning. Despite this, efforts were made to align each of the focus and sample position to similar values when switching between OCT systems.

The 1.3 µm and 4 µm OCT systems employ different sample interface optics with different numerical apertures (NA) of 0.09 and 0.23, respectively, determining different depth of focus intervals.

As chromatic aberrations are more notable for the 4 µm OCT system (seen when measuring the lateral resolution), this may explain why the depth of focus range is similar to the 1.3 µm system, of ~2 mm. Again, when characterizing a sample, efforts were made to position the focus in the same depth region of the sample when switching between OCT systems. Therefore, the influence of the different interface optics between the two OCT systems is considered negligible.


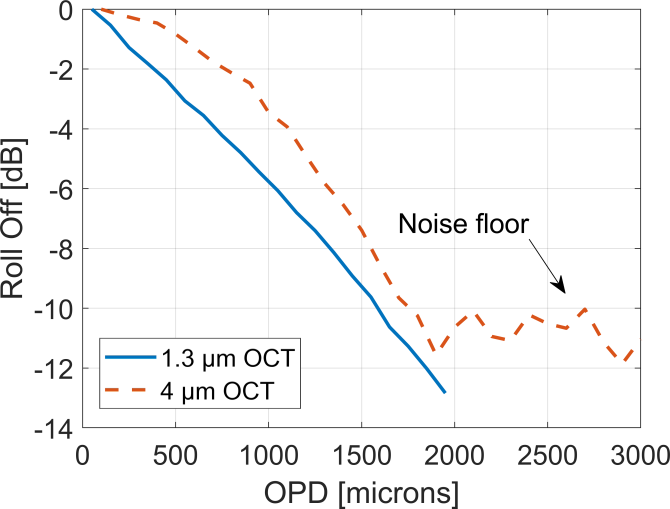


Fig. S6 Sensitivity roll-off of the 1.3 and 4 µm OCT systems.

In order to make the comparison of images meaningful, the color scale in each image is normalized to the maximum signal, with the minimum value being 1.2 times the mean noise value at OPD = 2 mm. The minimum value is to suppress the presence of speckle noise in the images.

When comparing the Monte-Carlo simulated OCT images, the upper contrast threshold (e.g. white) was fixed to the maximum image value and the lower contrast threshold (e.g. black) fixed to a value 30 dB less than the maximum image value.

1. **Direct comparison with state of the art**

To demonstrate the improvement over existing state-of-the-art, a direct comparison with results from ref. 19 and ref. 28 of the manuscript is presented.
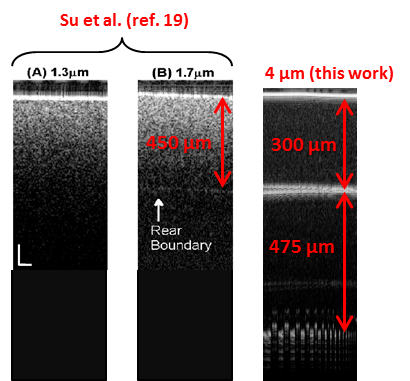

**Fig. S7 Comparison of B-scans of alumina reported by Su et al. (ref. 19 in the manuscript) and this work.**

Fig. S7 and S8 show a direct comparison of B-scans and C-scans obtained from ref. 19 and 28 of the manuscript, respectively, with the corresponding 4 μm B-scan and C-scan of the alumina plates reported in this work. Note that in this work imaging through both 300 μm and 475 μm alumina plates was demonstrated. It is clear that the 4 μm OCT system offers a vast improvement in penetration depth compared to the 1.7 μm OCT system.


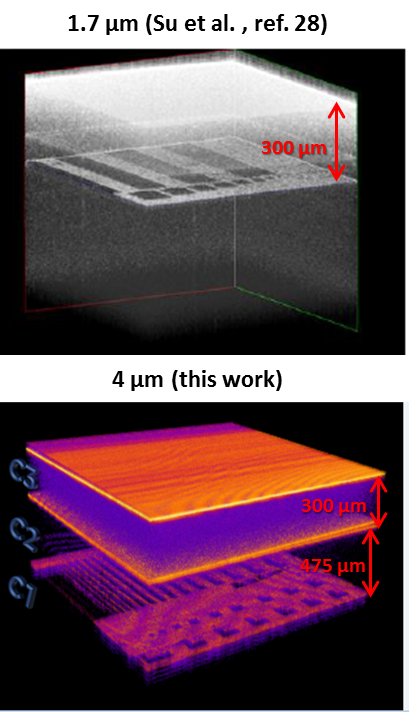
 **Fig. S8 Comparison of C-scans of alumina reported by Su et al. (ref. 28 in the manuscript) and this work.**
